# Engaging Informal Private Health Care Providers for TB Case Detection: Experiences from RIPEND Project in India

**DOI:** 10.1155/2021/9579167

**Published:** 2021-06-22

**Authors:** Santosha Kelamane, Srinath Satyanarayana, Sharath Burugina Nagaraja, Vikas Panibatla, Ramesh Dasari, Rajeesham, Amera Khan, Vishnuvardhan Kamineni

**Affiliations:** ^1^TB Alert India, Hyderabad, India; ^2^Independent Public Health Consultant, New Delhi, India; ^3^ESIC Medical College and PGIMSR, Bengaluru, India; ^4^State TB Office, Telangana State, Hyderabad, India; ^5^Stop TB Partnership, Geneva, Switzerland; ^6^Senior Advisor, Public Health, Bengaluru, India

## Abstract

**Background:**

Informal (unqualified) health care providers are an important source of medical care for persons with presumptive TB (PPTB) in India. A project (titled RIPEND) was implemented to engage informal providers for the identification of PPTBs and TB patients in 4 districts of Telangana State, India, during October 2018-December 2019 project period. Engagement involved sensitizing the informal providers about TB, providing them financial incentives to identify PPTBs, and linking these PPTBs to diagnostic and treatment services provided by the Government of India's National TB Elimination Programme.

**Objectives:**

To describe (a) the characteristics of the informal providers, along with their self-reported practices on TB diagnosis, treatment, and challenges encountered by the RIPEND project staff in engaging them in the project and (b) the outputs and outcomes of this engagement.

**Methods:**

We used a combination of one-on-one interviews with informal providers, group interviews with RIPEND project staff, and secondary analysis of data available within the project's recording and reporting systems.

**Results:**

A total of 555 informal providers were actively engaged under the project. The majority (87%) had a nonmedicine-related graduate degree and had been providing medical care for more than 10 years. Most (95%) were aware that a cough for 2 weeks or more is a symptom of pulmonary TB and that such patients should be referred for sputum-smear microscopy at a government health facility. Challenges in engaging the informal providers included motivating them to participate in the study, suboptimal mobile usage for referral services, and delays in providing financial incentives to them for referring PPTBs. During the project period (October 2018-December 2019), 8342 PPTBs were identified of which 1003 TB patients were detected and linked to TB treatment services.

**Conclusion:**

This project showed that engaging informal providers is feasible and that a large number of PPTB and TB patients can be identified through this effort. The Government of India should consider engaging informal providers for the early diagnosis of TB to reduce the missing TB cases in the country.

## 1. Introduction

India accounts for nearly 27% of the global tuberculosis (TB) burden [1]. Despite the efforts of the Government of India's Revised National TB Control Programme (RNTCP) over the past couple of decades, TB continues to be a major public health problem. The country has now renamed RNTCP to National TB Elimination Programme (NTEP) with a goal of reducing the TB burden drastically by 2025, five years ahead of the United Nations' Sustainable Development Goals (SDG). The road map for TB elimination is outlined in National Strategic Plan (2017-2025) [2].

For eliminating TB, it is essential to diagnose and treat all TB cases occurring in the community [3]. In countries like India, involvement of private health care providers is one of the quintessential processes to achieve TB elimination [4]. The private health sector comprises both formal and informal health care providers in almost equal proportions [5]. The formal private sector is relatively expensive, particularly for the economically poor and vulnerable groups. Therefore, they avail medical services from the informal private sector, which is more affordable and easily accessible [6]. In India, nearly 70% of the persons with presumptive TB (PPTB), especially those from low and middle-income communities, first approach a nearby private health care provider who is usually an informal provider [7–9]. It is sometimes thought that the quality of care is compromised by informal care providers and that this often leads to delayed diagnosis and inappropriate treatment [10]. However, it is important for a national health programme to have a greater accessibility and deeper penetrability in the community.

The Government of India's NTEP has made several efforts to engage the formal private health care providers [11, 12], but the efforts made to engage the informal providers have not been commensurate to the need. The main reason for hesitancy to engage informal providers is that these providers are technically unqualified and therefore illegal practitioners of medical care. Any effort to engage them is seen as a sign of acknowledging and legitimising their presence [13]. However, it is now being increasingly recognised that it is essential to engage informal providers for reaching out to all TB patients because of their widespread presence in all parts of the country and their deep connection with the lower socioeconomic strata of the population who are disproportionately affected by TB disease [2, 14]. There is sparse evidence in the country on successful models to engage these providers [12, 15].

TB Alert India, an international nongovernment organisation, is implementing a project called RIPEND (Roping Informal Private Providers for Enhanced TB case Detection) with funding support from the Stop TB Partnership's TB REACH Wave 6 grant [16] in the state of Telangana, India. The first phase of this project was implemented in rural and semiurban areas of 8 TB Units (covering a population of ~2.2 million people) with an aim of training and engaging informal private health care providers in early detection of TB among patients and referring them for health/medical care. Under this project, we mapped all the informal providers in the TB units, if they met certain eligibility criteria (the criteria are described in Methods). The engaged informal provider underwent a one-day training on the identification of PPTB, the referral of PPTB to undergo TB diagnostic evaluation as per the NTEP diagnostic algorithm, and the linkage of diagnosed TB patients to treatment services. The presumptive TB patients are subjected to sputum-smear examination and chest radiography for TB diagnosis. If the smear results are negative and chest X-ray suggestive of TB or not, the presumptive TB patient shall be subjected to cartridge-based nucleic acid amplification test (CB-NAAT) for the detection of *Mycobacterium tuberculosis* and rifampicin resistance. The patients who get the CBNAAT test (1) are smear positive and have chest X-ray suggestive of TB, (2) are smear positive but have chest X-ray not suggestive of TB, (3) are smear negative but have chest X-ray suggestive of TB, and (4) have high clinical suspicion (smear negative or unavailable and chest X-ray not suggestive of TB or unavailable).

As part of the RIPENED project, we examined (a) the sociodemographic characteristics of the informal providers, along with their self-reported practices on TB diagnosis and treatment, and the challenges encountered by the RIPEND project staff in engaging them in the project and (b) the outcomes of this engagement in terms of the number of PPTB identified, the numbers assessed for TB with various TB diagnostic tests, and the numbers of TB patients diagnosed and linked to TB treatment services under the NTEP.

## 2. Methods

### 2.1. Study Design

*Under objective 1*, we adopted a cross-sectional study design for assessing the sociodemographic characteristics of the informal providers, along with their self-reported practices on TB diagnosis and treatment. For assessing the challenges encountered in engaging informal providers, we conducted qualitative interviews with TB Alert program staff who were responsible for engagement activities. *Under objective 2*, for describing the outcomes of the engagement, we analysed the data collected on the persons with PPTB identified by the informal providers under the project.

### 2.2. Study Setting

TB Alert India implemented the RIPEND project in eight tuberculosis units (TUs) of four districts, Karimnagar, Jangaon, Jagtial, and Warangal in the State of Telangana. This project was implemented in close partnership with the District/State TB programme managers.

In all eight TUs, the field coordinators of the project (*n* = 16) had line listed all informal health care providers. Providers who met the following eligibility criteria mentioned were involved under the project: (a) provide consultations to at least 10 patients per day, (b) willing to participate in the project, and (c) have an android smart phone and are willing to use it for recording and reporting patient details. All the engaged providers were trained about the programme and the project, including usage of a mobile application (an application for usage in the smart android mobile phones) for presumptive TB patient referrals by the trained RIPENED project staff. A nominal financial incentive (INR 40 for PPTB identification and referral for testing under the project and an additional INR 250 if PPTB were to be diagnosed as TB) was provided to the providers for identifying the presumptive pulmonary TB patient (PPTB) during their routine outpatient consultations and entering the PTC into the project's IT platform via their smartphones.

### 2.3. Study Period

The study was conducted between October 2018 and December 2019.

### 2.4. Study Population, Sampling, and Sample Size

For the quantitative component of the first study objective, all the informal providers willing to be part of the project were enrolled for the study; for the qualitative component, we conducted group interviews with the staff of the RIPEND project (~20). For the second study objective, we included all persons with PPTB identified by the informal providers during the project period without any exclusion.

### 2.5. Recording and Reporting Systems under the Project

Project RIPEND has established a paper-based and a mobile (both Android and iOS applications) web-based IT platform for documenting the information on the informal providers enrolled in the project. For each PPTB enrolled into the project, the following was documented: (a) general characteristics of the patients and their presentation such as name, age, sex, address, symptoms, and their duration; (b) type of diagnostic tests and their results, such as sputum smear microscopy, chest radiography, and CB-NAAT such as Xpert MTB/Rif® tests; and (c) the basis of diagnosis for treatment initiation and the type of sector under which treatment was initiated. The data from this information technology platform was exported to Microsoft Excel and analysed. This database was used to generate the quarterly reports that were shared with the local TB programme managers and the funders. The validity/accuracy of the data contained in the recording and reporting system was periodically assessed by RIPEND project managers during routine supervisory visits and external TB REACH monitoring and evaluation visits. If any discrepancy was noted in the data, it was rectified immediately.

### 2.6. Data Variables, Data Collection, and Sources of the Data

For objective 1, we used a structured questionnaire to capture the following information from the informal providers: age, sex, qualification, number of years in the field, type of clinic, other facilities with clinic (pharmacy or laboratory), location (crowded place/isolated), building (rented/own), average consultation fees per patient, average time spent on each patient, whether the provider conducts home visits and does the provider conduct a mobile clinic, TB diagnostic practices (referral for sputum/chest X-ray/both/others), diagnosis of TB made by self/other qualified professionals and diagnostic tests used, TB treatment practices (NTEP drugs/private drugs from pharmacies) anti-TB drugs/others-additional/both/self-made preparation, willingness to update his knowledge/undergo training on TB (yes/no), time desirable to spend on knowledge improvement (1 hr/4 hr/7 hr), preferred day in a week (week days/weekends), mode of knowledge improvement (pamphlet/mobile messages/one-to-one discussion/class room teaching/others), and frequency of knowledge improvement (monthly/quarterly).

We used an interview guide to conduct group interviews with our project staff to collect the information on the challenges encountered in engaging informal care providers (like training, enrolment, and continuous engagement of informal providers and sustainability). The key informant interviews were conducted by trained researchers in the local language (Telugu) at a place and time convenient to them. Interviews were audio recorded. The transcripts were translated into English and used for analysis. We ensured the accuracy of translation through the translation-back translation method.

For objective 2, from the project's mobile app-based recording and reporting system, we obtained the following data for each PPTB: their demographic characteristics include age, sex, type, and duration of symptoms with a specific focus on cough, type of diagnostic tests, and test results (sputum smear, chest radiography, and CB-NAAT); if TB were diagnosed, type of TB (new smear-positive, new smear-negative, retreatment smear-positive, and rifampicin-resistant TB), source of TB treatment (public/private), and the TB notification status to NTEP were recorded.

### 2.7. Data Analysis

For objective 1, the quantitative data were entered in a standard data entry software (EpiData, Odense, Denmark) and were analysed using EpiData analysis software. For the qualitative component of the data, the content analysis of the transcripts was done. The two study investigators (SS and SBN) independently identified all codes and generated themes. Then, the codes and themes were compared, and the differences were sorted out through consensus and detailed discussions. The verbatim quotes to support the codes and themes were used to describe the results of the qualitative study.

For objective 2, we imported the data from Microsoft Excel and analysed the data in Stata Statistical Software (version 15, Stata Corp, College Station, Texas, US). We have summarized the various patient characteristics by numbers and proportions.

### 2.8. Ethics Approval

Ethics approval for the study was obtained by the Institutional Ethics Committee of National TB Institute, Bengaluru, Karnataka. We obtained informed consent from all study participations under objective 1. We got a waiver from obtaining informed consent from the study participants under objective 2. We have maintained the confidentiality of the data throughout the study.

## 3. Results

### 3.1. Sociodemographic Characteristics

Under objective 1, a total of 555 informal providers were enrolled in the study. The sociodemographic characteristics of the practitioners are shown in Table 1. The majority of them were in the age group of more than 40 years (60%) and were males (99%). The highest number of providers was enrolled from the Jagtial TU (21%), followed by Warangal DTC TU (20%) and Karimnagar DTC TU (18%). The majority (82%) of the providers had completed graduate-level education in a nonmedicine-related field and had more than 10 years of experience; 50% of them had clinics in rented buildings. Most of the clinics operated during both the mornings and evening hours; the majority catered to about 10-20 out-patient attendees per day. They charged the patients anywhere between 0.5 and 1 USD per consultation.

### 3.2. Tuberculosis Management Practices

The TB management practices of the providers at the baseline are shown in Table 2. They revealed that the majority (95%) of the providers were aware that a cough of two weeks or more is the predominant symptom of TB disease, and for persons with such symptoms, most are prescribed sputum smear examination at a government health facility. None of them preferred to order chest radiography or blood tests as the first choice of investigation for TB disease. Nearly 84% of them were sensitized on TB by the project's personnel. Few of the providers (4%) were also treatment supporters or DOT providers under NTEP.

About 40% of the practitioners were using the mobile application for enrolling PPTBs under the project. The remaining were using a logbook (provided by the project) to maintain the PPTB details from which data were subsequently entered into the mobile application. After six months of initiating the project, 85% of the providers mentioned that they were receiving the incentives on time for referring the presumptive cases or TB patients. Most of the providers were willing to undergo at least four hours of updated training.

### 3.3. Challenges to Engage Informal Care Providers: Perspectives of Project Staff

During group interviews, the challenges faced by the staff in engaging with the informal providers are listed in Table 3. The challenges were predominantly related to sensitization, referral activities, and financial incentives. Most of the project staff opined that it is essential to engage the informal health care providers under the national TB programme, as they provide medical care to a significant proportion of the population.

“…*it is difficult to mobilise them [informal providers] for sensitization, though they are interested, they don't want to lose their livelihood…”* Male, 33 years, Karimnagar DTC TU.

“...*some [informal providers] don't have smart phones and those who have it use it for basic services like phone calls and whatsapp!...we have to be behind them every day to make them refer patients through mobile application…very difficult!”* Male 28 years, Station Ghanpur TU.

“…*though incentive is not of great attraction to them [informal providers]…at times…they complain about not receiving it!...they expect it to happen immediately…how is it possible?...”* Male, 42 years, Mulkanoor TU.

### 3.4. Clinical-Demographic Features of Referred Presumptive TB Patients

During the project period, approximately 8342 PPTBs were identified and referred for testing. Their demographic and clinical characteristics are presented in Table 4. Nearly 44% of the patients were in the age group of 18-45 years, 63% were males, and 92% had cough as the presenting symptom with 85% having cough for 2 to 3-week duration. Of all the presumptive TB cases screened, 54% of them knew their HIV status and 0.6% of them were found to be HIV positive. Nearly 3.5% of the persons had a past history of TB.

### 3.5. Diagnostic Tests Undergone by the Presumptive Cases

The diagnostic tests undergone by the 8342 persons with presumptive pulmonary TB are given in Table 5. Approximately 10% of the persons with presumptive pulmonary TB had undergone X-ray, of which 32% had X-ray findings suggestive of TB. Approximately two-thirds of the persons with presumptive TB had undergone sputum smear examination, of which 6.5% had sputum smear-positive results. Approximately a third of the persons with presumptive TB had undergone CB-NAAT tests, and of these who had taken this test, 23% had a positive result, indicating the presence of TB bacilli.

### 3.6. Notification to the Public Health System

Of the total 8342 persons with presumptive TB patients, 1003 cases of TB were diagnosed, data that were communicated to the public health systems. The testing pattern of TB diagnostic tests performed, the number of patients who underwent tests, and those diagnosed with TB are shown in Table 6. The number and proportion of patients subjected to only a single diagnostic test were (a) microscopy (4785, 57%), (b) CB-NAAT (1984, 24%), and (c) chest X-ray (235, 3%), while the number and proportion of patients who underwent two diagnostic tests are (d) microscopy and chest X-ray (235, 3%), (e) microscopy and CB-NAAT (431, 5%), and (f) chest X-ray and CB-NAAT (223, 3%). The patients who underwent (g) microscopy and chest X-ray and CB-NAAT were 98 (1%).

Similarly, the number and proportion of TB patients who were diagnosed based on single diagnostic tests were (a) microscopy (201, 20%), (b) CB-NAAT (29, 3%), and (c) chest X-ray (417, 42%). The number and proportion of patients who underwent two diagnostic tests were (d) microscopy and chest X-ray (44, 4%), (e) microscopy and CB-NAAT (150, 15%), and (f) chest X-ray and CB-NAAT (104, 10%). The patients who underwent (g) microscopy and chest X-ray and CB-NAAT were (58, 6%).

### 3.7. Clinical-Demographic Features of Diagnosed TB Cases

The TB detection rate among persons with presumptive TB disaggregated by their demographic and clinical status is given in Table 7. Within different age groups, TB detection ranged from 10% in those aged less than 18 years to 15% in those aged 18-45 years. In males, 14% were detected with TB when compared to 9% among females. 13% of those who had a cough were diagnosed with TB. In those that were HIV positive, 32% were detected with TB when compared to 12% of those whose HIV status was unknown. The previous history of TB was detected among 33% of patients when compared to 12% among those without a past history of TB.

## 4. Discussion

The study describes the process, challenges, outputs, and outcomes of a project that was aimed towards engaging the informal providers. Through this project, ~555 informal providers were engaged, and through them, 8342 PPTBs were identified and subjected to diagnostic tests, of which 1003 persons were diagnosed with TB and all the patients were initiated on treatment according to programme guidelines. Sputum smear microscopy was the major diagnostic test for TB case detection.

The study findings suggest that the majority of the informal health care providers are educated, have knowledge of TB management practices, and can be engaged in the detection of TB patients in the community. The major challenges faced by the project staff were motivating the rural practitioners to participate in the study, suboptimal mobile usage for referral services, and delayed financial incentives for the referred presumptive TB patients.

The study has the following programmatic implications. First, informal providers can be successfully engaged, and sustained efforts will result in increasing presumptive identification and promote early TB diagnosis, particularly improving TB care among the vulnerable groups. Despite being technically unqualified to provide health care services, they had been providing the basic services in their locality for many years; hence, the NTEP should seriously consider engaging the informal providers (through training, incentives) for early identification of PPTBs by building their capacity and to accept referrals to government health services for TB management. [2, 14, 17]. A similar finding was observed in a study conducted at Jharkhand by Project Axshya [18]

Second, the informal providers were given nominal financial incentives under the project to encourage them to identify and refer PPTB for testing. We strongly believe that this incentive played a role in their engagement. Although there were instances of delayed disbursement of incentives for the providers under the project, it did not deter the identification and referral of PPTBs from the providers. The NTEP has provisions for providing incentives to the private health care providers for diagnosis of TB [12]. This should be extended for identification and referral of PPTBs as well.

Third, engagement of informal providers resulted in the identification of large numbers of PPTB, roughly constituting 7-8% of the TB patients detected in these TB Units [19]. The yield in the absolute number of PPTBs and TB cases was higher than a similar project conducted in Odisha State of India [20]. In the absence of this project, the 8342 PPTBs identified under this project either would have not been subjected to TB diagnostic evaluation or would have been subjected to diagnostic evaluation at a later stage. This would have resulted in missed diagnosis or delayed diagnosis [21]. This would have increased the morbidity, mortality among these individuals, and the transmission of TB in the community, which was perhaps mitigated by the implementation of this project [20].

Fourth, based on anecdotal information obtained by the project's field coordinators, we feel that 8342 persons with TB symptoms identified under the project do not represent all cases of presumptive TB, and therefore, the number of persons with TB symptoms identified under this project is a gross underestimate of the actual number of presumptive TB patients seeking care from them. In our project, we tried to enhance the identification of presumptive TB patients by interacting with the informal providers at regular intervals through field coordinators and also provided a token financial incentive for identification and referral of presumptive TB cases through mobile application. But it appeared to be insufficient, and several other steps may have to be undertaken to enhance the completeness of referrals by informal health care providers, probably because the field coordinators felt that the informal providers still weighed the economic condition of the PPTBs and referred them to private health care facilities that perhaps gave them higher kick-backs. Lesser technical skills of informal providers to elicit proper history and to identify atypical presentations of PPTB [22] are a limitation. Identifying other strategies is an area for future operational research.

Fifth, there were several gaps in diagnostic tests undergone by PPTBs enrolled under the project, especially with respect to X-ray testing, sputum smear microscopy, and CB-NAAT testing. The reasons for these gaps appear to be nonavailability of these tests at the local level and difficulties in accessing these tests at the site of their availability. The project tried to mitigate these problems by establishing sputum collection and transportation systems (for sputum smear microscopy and CB-NAAT tests), identifying X-ray facilities, and linking patients to these facilities. Despite these measures, the gaps remained.

Sixth, the TB detection rate was 12% among all the presumptive TB patients enrolled in this project. This is comparable to the TB positivity under the NTEP [19]. Though this is a significant proportion, we feel that it is an underestimate, given that all persons with presumptive TB did not undergo a full diagnostic evaluation as per the NTEP diagnostic algorithm. Therefore, the true magnitude of TB cases among these PPTB is unknown. Also, as expected, the TB detection rates were higher in certain high-risk groups (persons who were HIV positive, persons with a past history of TB, and those who had a cough of longer duration) as shown in Table 7.

Lastly, anecdotal evidence suggests that there is a lack of trust in the public health system, preference for treatment under the care of private health care providers (despite incurring out-of-pocket expenditure), and the fear that accessing treatment from the public health facilities may affect the confidentiality of the disease similar to what has been reported in other studies [8, 9]. More work needs to be done for addressing these concerns and to help patients access treatment from public health facilities.

The major strength of study is that it was conducted under programmatic settings and reflects reality in the field. The major limitation of this study is the fact that the referral of TB cases by the informal care providers was incentivized and the sustainability of the referrals after the project period is unknown.

To conclude, this project demonstrated that engaging informal health care providers is feasible. Keeping informal providers updated about availability and accessibility of TB services in their areas is of paramount importance, and having robust mechanisms to monitor informal providers will lead to better TB case detection. However, this study also showed that there are several challenges that need to be addressed; the most prominent ones are ensuring completeness in the identification of persons with presumptive TB; ensuring that all persons with presumptive TB undergo complete diagnostic tests as per the NTEP's algorithm; and finally ensuring that all TB patients are treated under the public health system.

## Figures and Tables

**Table 1:** Sociodemographic characteristics of informal providers involved in RIPENED project, Telangana State, India, 2018-19 (*N* = 555).

S. no	Variables		Number (%)
1	Age group (years)	<40	216 (39)
		40-50	204 (37)
		51-60	100 (18)
		>60	35 (6)

2	Sex	Male	551 (99.5)
		Female	4 (0.5)

3	Tuberculosis unit	Mulkanoor	69 (12)
		Station Ghanpur	71 (13)
		Warangal DTC	111 (20)
		Choppadandi	44 (8)
		Karimnagar DTC	99 (18)
		Jangaon	46 (8)
		Jagtial	115 (21)

4	Area of clinic	Urban	486 (87)
		Rural	69 (13)

5	Qualification	High school	26 (5)
		10^th^ standard	36 (6)
		12^th^ standard	8 (1)
		Diploma degree	196 (35)
		Degree science	187 (34)
		Degree others	102 (18)

6	Health facility	Rented	277 (50)
		Own building	267 (48)
		Not recorded	11 (2)

7	Clinic timings	Morning	1 (0)
		Morning and evening	546 (97)
		Evening	6 (3)

8	Experience in medical practice (in years)	<10	101 (18)
		10-20	240 (43)
		20-30	152 (28)
		>30	62 (1)

9	No. of daily consultations	<10	4 (0)
		10-20	469 (85)
		20-30	80 (14)
		>30	4 (1)

10	Consultation charges (INR)	50	21 (4)
		50-75	524 (94)
		>75	10 (2)

**Table 2:** TB management practices of Informal providers at the time of involvement under RIPENED project, Telangana State, India, during 2018-19 (*N* = 555).

S. no	Variables		Number (%)
1	Symptoms of presumptive TB	Cough ≥ 2 weeks	528 (95)
		Not recorded	27 (5)

2	Action taken when encountered presumptive TB	Referred	1 (0.5)
		Order TB test	553 (99)
		Not recorded	1 (0.5)

3	Where do you refer?	Government	555 (100)
		Private	0

4	Do you ask for chest radiography?	Yes	0
		No	552 (99)
		Not recorded	3 (1)

5	Do you ask for sputum smear microscopy?	Yes	553 (100)
		No	0
		Not recorded	2 (0)

6	Do you ask for blood test?	Yes	0
		No	554 (99)
		Not recorded	1 (1)

7	Are you sensitized on TB by project staff?	Yes	466 (84)
		No	87 (16)
		Not recorded	2 (0)

8	Where were you sensitized?	Government	454 (82)
		Private	7 (3)
		Not recorded	94 (17)

9	Are you a DOT provider?	Yes	25 (4)
		No	518 (94)
		Not recorded	12 (2)

10	Do you use project mobile application routinely?	Yes	218 (40)
		No	330 (60)
		Not applicable	2 (0)
		Not recorded	5 (1)

11	Have you received incentives from project for referrals? (Initial 3 months)	Yes	86 (15)
		No	466 (85)
		Not recorded	3 (0)

12	Are you willing to undergo programmatic sensitization?	Yes	528 (95)
		No	16 (3)
		Not applicable	3 (1)
		Not recorded	8 (1)

13	How many hours can you devote for sensitization?	4 hours	537 (97)
		1 day	1 (0)
		Not recorded	17 (3)

**Table 3:** Key challenges encountered by the RIPENED project staff.

During sensitization (i) Availability of informal providers in a timely manner for group sensitization programs (ii) Incompatible smart phones of informal providers to support the project mobile application

During referral activities (i) Inefficient usage of mobile application for referral activities (ii) Frequently deleting the mobile application from their phones (iii) Referring the symptomatic to private hospitals (iv) Referring of symptomatic with less than one-week cough to sputum smear microscopy (v) Inadequate emphasis of chest x-ray on patients when smear results are negative and symptoms are present

Financial incentives (i) The providers perceived the incentive provided as low (ii) Delays in disbursement of funds to the providers bank accounts

**Table 4:** Characteristics of persons with presumptive TB enrolled under RIPENED project, Telangana State, India, during 2018-19 (*N* = 8342).

S. no	Characteristics	Number	(Percent)
1	Age group		
	<18	378	(4)
	18-45	3604	(44)
	>45 to 60	2636	(32)
	>60	1724	(20)

2	Gender		
	Female	3079	(37)
	Male	5260	(63)
	Transgender	3	(0)

3	Cough		
	Yes	7688	(92)
	Unknown	573	(7)
	No	81	(1)

4	Cough (duration), weeks		
	<2	207	(3)
	2-3	6627	(85)
	≥4	935	(12)

5	HIV status		
	Positive	53	(0)
	Negative	4464	(54)
	Unknown	3252	(39)
	Not recorded	573	(7)

6	Previous history of TB		
	Yes	289	(3)
	No	7480	(90)
	Unknown/not recorded	573	(7)

**Table 5:** Results of different TB diagnostic tests of Presumptive TB patients enrolled under RIPENED project, Telangana State, India, during 2018-19 (*N* = 8342).

S. no	Characteristics	Number	(%)
1	Sputum microscopy		
	Not done	2793	(33)
	Done	5549	(67)

2	Microscopy results (if done)		
	Negative	5186	(93)
	Positive	363	(7)

3	Chest X-ray result		
	Not done	7551	(90)
	X-ray done	791	(10)

4	X-ray result (if done)		
	Not suggestive of TB	537	(68)
	Suggestive of TB	254	(32)

5	CB-NAAT test		
	CB-NAAT test not done	5606	(67)
	CB-NAAT test done	2736	(33)
	CB-NAAT result (if done)		
	MTB not detected	2073	(76)
	CB-NAAT result not available	33	(1)
	MTB detected-RIF sensitive	512	(19)
	MTB detected-RIF resistant	115	(4)

**Table 6:** The testing patterns of TB diagnostic tests performed, number of patients who underwent tests and those diagnosed with TB under RIPENED project, Telangana State, India, during 2018-19 (*N* = 8342).

Testing patterns of TB diagnostic tests	Number of patients who underwent the test (*N* = 8342) *n* (%)	Number diagnosed with TB (*N* = 1003) *n* (%)
(a) Microscopy only	4785 (57)	201 (20)
(b) Chest X-ray only	235 (3)	29 (3)
(c) CB-NAAT only	1984 (24)	417 (42)
(d) Microscopy+chest X-ray only	235 (3)	44 (4)
(e) Microscopy+CB-NAAT only	431 (5)	150 (15)
(f) Chest X-ray+CB-NAAT only	223 (3)	104 (10)
(g) Microscopy+chest X-ray+CB-NAAT	98 (1)	58 (6)
(h) No tests done	351 (4)	0 (0)

**Table 7:** Proportion of TB cases detected among different characteristics of presumptive TB patients under RIPENED project, Telangana State, India, during 2018-19 (*N* = 8342).

S. no	Characteristics	Presumptive TB patients	TB patients	(%)
1	Age group			
	<18	378	38	(10)
	18-45	3604	499	(15)
	>45 to 60	2636	306	(13)
	>60	1724	160	(12)

2	Gender			
	Female	3079	287	(9)
	Male	5260	716	(14)
	Transgender	3	0	(0)

3	Cough			
	Yes	7688	959	(13)
	Unknown	573	32	(6)
	No	81	12	(15)

4	Cough (duration), weeks			
	<2	207	18	(8)
	2-3	6627	667	(10)
	≥4	935	266	(28)

5	HIV status			
	Positive	53	17	(32)
	Negative	4464	586	(13)
	Unknown	3252	368	(11)
	Not recorded	573	32	(6)

6	Previous history of TB			
	Yes	289	94	(33)
	No	7480	877	(2)
	Unknown/not recorded	573	32	(6)

## Data Availability

The data used to support the findings of this study are available from the corresponding author upon request and permission from TB Alert, India.
